# ABA and SA Participate in the Regulation of Terpenoid Metabolic Flux Induced by Low-Temperature within *Conyza blinii*

**DOI:** 10.3390/life13020371

**Published:** 2023-01-29

**Authors:** Ming Yang, Maojia Wang, Ming Zhou, Yifu Zhang, Keliang Yu, Tao Wang, Tongliang Bu, Zizhong Tang, Tianrun Zheng, Hui Chen

**Affiliations:** 1 Traditional Chinese Medicine Planting Institute, Chongqing Academy of Chinese Materia Medica, Chongqing College of Traditional Chinese Medicine, Chongqing 400065, China; 2 College of Life Science, Sichuan Agricultural University, Ya’an 625014, China; 3 YongChuan District Center for Disease Control and Prevention, Chongqing 402160, China

**Keywords:** *Conyza blinii* (Jin Long Dan Cao), low-temperature stress, terpenoid metabolic flux, phytohormone fluctuation

## Abstract

Under dry-hot valley climates, *Conyza blinii* (also known as Jin Long Dan Cao), suffers from nocturnal low-temperature stress (LTS) during winter. Here, to investigate the biological significance of terpenoid metabolism during LTS adaptation, the growth state and terpenoid content of *C. blinii* under different LTS were detected, and analyzed with the changes in phytohormone. When subjected to LTS, the results demonstrated that the growth activity of *C. blinii* was severely suppressed, while the metabolism activity was smoothly stimulated. Meanwhile, the fluctuation in phytohormone content exhibited three different physiological stages, which are considered the stress response, signal amplification, and stress adaptation. Furthermore, drastic changes occurred in the distribution and accumulation of terpenoids, such as blinin (diterpenoids from MEP) accumulating specifically in leaves and oleanolic acid (triterpenoids from MVA) accumulating evenly and globally. The gene expression of MEP and MVA signal transduction pathways also changes under LTS. In addition, a pharmacological study showed that it may be the ABA-SA crosstalk driven by the LTS signal, that balances the metabolic flux in the MVA and MEP pathways in an individual manner. In summary, this study reveals the different standpoints of ABA and SA, and provides a research foundation for the optimization of the regulation of terpenoid metabolic flux within *C. blinii*.

## 1. Introduction

Perennial plants usually need to be exposed to a continuous cold environment to achieve the purpose of flowering, which is called vernalization. Related genes are expressed programmatically under specific conditions to regulate this complex process [1,2]. Glucose, nucleic acid, and protein metabolism play a key role in vernalization [3]. 

The balance between plant resistance and growth under low temperatures occurs at multiple equal levels. A variety of biological processes are used to maintain the balance between stress resistance and growth at low temperatures [4]. Molecular and physiological responses of plant temperature perception are closely related to the regulation of phytohormone which maintain the stability of plant immunity, the stress response, and the development process in the process of temperature change [5]. Early signaling of Ca^2+^ and the SNF1-related protein kinases (SnRKs) transmit low-temperature signals upstream of signal transduction, which plays a key role in the downstream abscisic acid (ABA) signaling pathway [6]. The OST1 kinase protein, as an important regulator in the ABA signaling pathway, regulates the expression of the *CBF* gene and plant cold tolerance through phosphorylation modification and by stabilizing ICE1 protein activity [7]. The cis element transcription factor HSFA1 (heat stress transcription factor A1) is activated by the salicylic acid (SA) receptor NPR1 (nonexpresser of *PR* genes 1) under low-temperature conditions, and regulates the expression of the *COR* gene through non-CBF pathways to regulate plant tolerance of low temperatures [8,9]. 

The cold stress signal pathway is closely related to other signaling pathways in the plant response under low temperatures. A low-temperature signal triggers plant hormone signals and stimulates more biological pathways to help plants to respond [10]. It was found that different cultural temperatures would affect the growth and differentiation ability of *Panax ginseng* adventitious roots. After stimulating adventitious roots under low temperatures for a certain time, the accumulation of ginsenosides increased with the expression of related genes [11]. The increase in artemisinin content in *Artemisia annua* under low-temperature stress is greater than under salt, water, low-temperature, and drought stress [12]. During the growth of *Taxus wallichiana* var. *mairei*, the highest content of paclitaxel is found in winter, which may help the plant to survive under a harsh environment [13].

As a biennial herb, *Conyza blinii* (*C. blinii*) needs vernalization to blossom and reproduce, and it also has been proven to have good medicinal value [14]. Terpenoids are not only the main medicinal components of *C. blinii* [15], but also their main tools to combat environmental changes [16]. Combining the chemical composition with growth development characteristics, we carried out relevant research on the terpenoids under low temperature within *C. blinii*. According to the morphological changes, basic metabolism, terpenoid metabolism, plant hormones, and other aspects of *C. blinii*, the physiological changes of *C. blinii* in the process of simulated vernalization were preliminarily explored. Our experiment lays a foundation for the understanding vernalization within *C. blinii.*

## 2. Materials and Methods

### 2.1. Plant Materials

The seeds and cultivation management methods of *C. blinii* were mentioned in our previous research [17]. When the plants grew to 2 months old, they were treated as samples with LTS and other experiments. Three independent biological repeats were set for each group. The real leaf, tender stem, and main root were selected for testing Location: Sichuan Agricultural University, Ya’an, China.

### 2.2. Low-Temperature Stress (LTS) Treatment

Firstly, in the nocturnal LTS experiment, plant materials were placed at 4 °C from 17:00 to 9:00 for 9 weeks and collected at 9:00 every other week. Secondly, for plant tissue under LTS, at a ground low temperature (Glt), heating bags were used to maintain the soil temperature at 25 ± 2 °C; for an underground low temperature (Ult), ice bags were used to maintain the soil temperature at 4 ± 2 °C. For the whole low temperature (Wlt), whole plants were treated at 4 ± 2 °C. Samples were collected after 0 h, 6 h, 12 h, 24 h and 48 h. All of these plants were managed under a long-day photoperiod (16 h: 8 h, light: dark). The relative humidity of the laboratory was 50–70%.

### 2.3. Treatment of Exogenous ABA, SA, and Their Inhibitors

The phytohormones and inhibitors involved in this study are referred in Yang et al. [18]. The concentrations of ABA/SA and ABT/FDT were 2 and 10 mM. Four-month-old *C. blinii* plants were selected in the above experiments. On the basis of ensuring the plant growth level, four-month-old *C. blinii* plants were cultured in 1/2-strength Hoagland’s solution for 7 days. Phytohormone and inhibitor solutions were added to the roots in 1/2-strength Hoagland’s solution. Samples were collected after 48 h. 

### 2.4. Analysis of Chlorophyll and Carotenoid Content

Refer to Plant Chlorophyll Content Test Kit (Beijing Solarbio Science and Technology Co., Ltd., Beijing, China, BC0995). The absorbance value was measured at 645 nm (chlorophyll A absorption peak) and 663 nm (chlorophyll B absorption peak) for the experimental operation. Refer to Plant Carotenoid Content Test Kit (Beijing Solarbio Science and Technology Co., Ltd., Beijing, China, BC4330) for experimental operation.

### 2.5. Content Detection of Cellulose and Pectin

Content detection of cellulose: Refer to Plant Cellulose Content Test Kit (Beijing Solarbio Science and Technology Co., Ltd., Beijing, China, BC4280) for experimental operation. Content detection of pectin: Refer to Plant Total Pectin Content Test Kit (Beijing Solarbio Science and Technology Co., Ltd., Beijing, China, BC4280) for experimental operation. 

### 2.6. Glandular Trichome (GTs) and Deciduous Leaf Counting

Referring to Zhan [19], GTs were counted after dyeing with rhodamine B. Leaves that were yellow, curly and shed from tissues were defined as deciduous leaves. Deciduous leaves were collected every other week.

### 2.7. Content Detection of Blinin and Oleanolic Acid 

In this section, the relevant experimental principles and detection methods for the content detection of blinin and oleanolic acid were in accordance with our previous research [17].

### 2.8. Content Detection of SA, ABA, IAA, GA_3_, ZT and BR

Samples were ground into powder under liquid nitrogen and extracted with methanol overnight at 4 °C [20,21]. Biological reference standards, i.e., zeatin (ZT), gibberellin (GA_3_), indole-3-acetic acid (IAA), abscisic acid (ABA), salicylic acid (SA) and brassinolide (BR) were all obtained from Shanghai Yuanye Biotechnology Co., Ltd, Beijing, China. Detection of HPLC: mobile phase—V_methanol_:V_0.6%_ acetic acid = 50%:50%. Column temperature—35 °C; flow rate—1.0 mL/min; injection volume—10 μL; detection wavelength—254 nm.

### 2.9. Screening and Expression Detection of ABA/SA Signal Transduction Pathway Genes

ABA/SA signal transduction pathways were assessed according to KEGG map04075 (https://www.kegg.jp/pathway/map04075, accessed on 20 January 2023). Screening of SA/ABA-related genes (CbNPR1, CbTGA, CbPR-1, CbPYL, CbPP2C, CbABF) was performed using the *C. blinii* transcriptome database [22] (accession number: PRJNA563166). The internal reference genes and related methods used in fluorescent qRT-PCR referred to our previous research [18,23,24]. Primer3 Plus (https://www.primer3plus.com/, accessed on 20 January 2023) was used to design fluorescent quantitative primers for genes online, and the detection fragment size was set to 150–250 bp.

### 2.10. Statistical Analysis and Illustration

Using the R Program (V 4.1.1) and Hmisc (V 4.6-0) toolkit, we carried out a statistical analysis. All the figure illustrations were obtained using Excel 2016 Home Edition and Cytoscape (V3.9.0). 

## 3. Results

### 3.1. The Change in the Basic Physiology of C. blinii during Nocturnal LTS 

**Figure 1:**
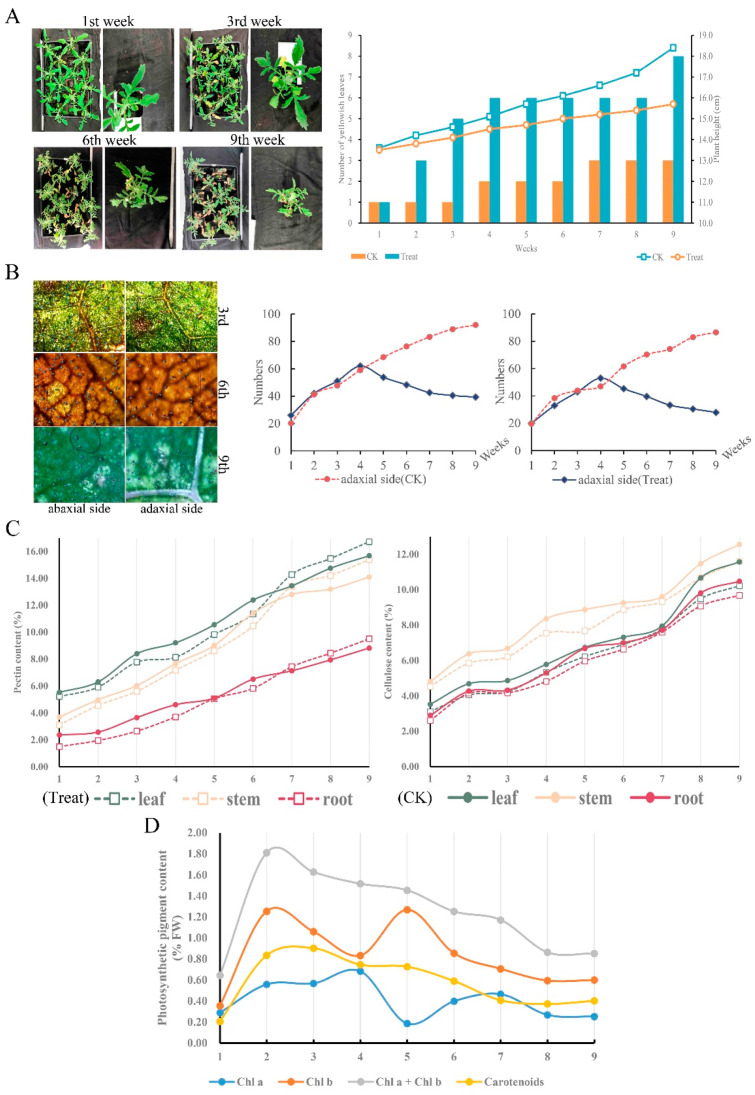
The morphology and characteristics of *C. blinii* during nocturnal LTS. *C. blinii* was subjected to nocturnal LTS for 9 weeks, and then the morphology and defoliation (**A**), the number of active GTs (**B**), the pectin and cellulose content in each tissue (**C**), and the photosynthetic pigment content (**D**) were detected. The CK group did not receive nocturnal LTS treatment.

To determine the physiological performance of *C. blinii* when suffering from nocturnal LTS, we observed and measured the leaf morphological characteristics and basic physiological indicators. Apparently, nocturnal LTS aggravated defoliation and suppressed stem growth severely (Figure 1A,B). Compared to the control group, the number of GTs on both the abaxial and adaxial surfaces maintained a relatively normal growth rate in the previous 4 weeks, but was reduced by more than half after being subjected to nocturnal LTS for 9 weeks (Figure 1B). Morphogenesis was affected under LTS; however, the content of cellulose and pectin increased smoothly, even exceeding that of the control group (Figure 1C). The determination results of photosynthetic pigment content showed that the content curves of chla, chlb, and car were similar to the parabola, and the content at the end of the study period was higher (Figure 1D).

### 3.2. The Accumulation Mode of Oleanolic Acid and Blinin in C. blinii during Nocturnal LTS

**Figure 2:**
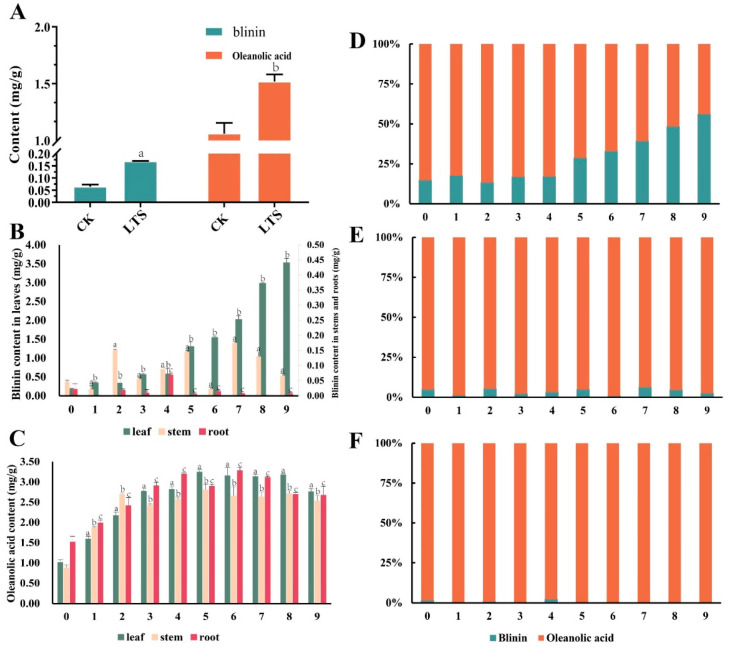
The content and distribution of blinin and oleanolic acid within *C. blinii* tissues during nocturnal LTS. After being subjected to nocturnal LTS for 1 week, an obvious accumulation occurred regarding blinin and oleanolic acid within leaves (**A**). During monitoring of the 9 weeks of nocturnal LTS, blinin displayed a specific distribution within leaves (**B**), while oleanolic acid displayed an even distribution (**C**). In order to observe the balance between MVA and MEP metabolic pathways, we calculated the percentage of blinin and oleanolic acid in leaves (**D**), stems (**E**) and roots (**F**) respectively. Three independent strains were selected. Different letters indicate significant differences between CK and experimental groups at the same time (a, b, c, *p* < 0.05).

In this study, the content of oleanolic acid was regarded as the metabolic flux of the MVA pathway, while the content of blinin was regarded as the metabolic flux of the MEP pathway. First of all, we found that the content of blinin and oleanolic acid with LTS was evidently higher than that without LTS (Figure 2A). Before plants were subjected to nocturnal LTS, the content of oleanolic acid was 1.01 mg/g, 0.88 mg/g, and 1.51 mg/g, respectively within the leaves, stems, and roots (Figure 2C). Due to the enhanced metabolism stimulated by LTS, the accumulation of oleanolic acid increased gradually, and the final content was 2.76 mg/g, 2.53 mg/g, and 2.67 mg/g, respectively, within the leaves, stems, and roots. Interestingly, between the 5th and 6th weeks, the content of oleanolic acid in the leaves, stems, and roots reached a peak. Unlike the above accumulation mode of oleanolic acid, blinin accumulated in leaves. As nocturnal LTS continued into the 9th week, the content of blinin increased from 0.17 mg/g to 3.52 mg/g, and the net increase was approximately two folds higher than that of oleanolic acid within leaves (Figure 2B). Unfortunately, it was difficult to detect the content range of blinin, which was 0.02–0.15 mg/g and 0.0014–0.07 mg/g, respectively within the stems and roots. When considering blinin and oleanolic acid as the total terpenoid metabolic flux, the MVA pathway and oleanolic acid seemed to dominate the plants, but MEP and blinin recaptured, step by step, more than half of the leaves (Figure 2D–F).

### 3.3. The Phytohormone Fluctuations in C. blinii during Nocturnal LTS

**Figure 3:**
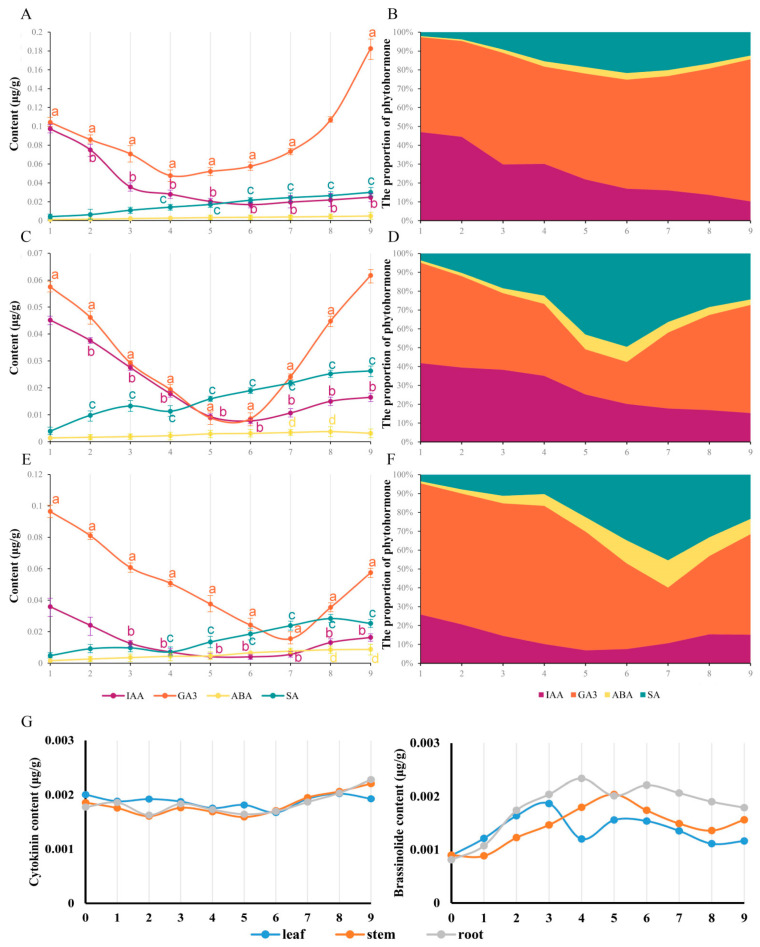
The content and distribution of phytohormones within *C. blinii* tissues during nocturnal LTS. We monitored and analyzed the trend of phytohormone content in roots (**A**), stems (**C**) and leaves (**E**) from 1 to 9 weeks of nocturnal LTS, and calculated the percentage of phytohormones (**B**,**D**,**F**). Unexpectedly, the contents of ZT and BR in this study were one order of magnitude lower than that of other plant hormones, so we independently displayed the trend (**F**,**G**). Three independent strains were selected. Different letters indicate significant differences between CK and experimental groups at the same time (a, b, c, *p* < 0.05).

As the result of the osmotic and oxidative stress induced by LTS, phytohormones participated in the occurrence and development of responses, especially auxin (IAA), gibberellin (GA3), abscisic acid (ABA) and salicylic acid (SA), with obvious fluctuations in this study, while brassinolide (BR) and cytokinin (ZT) were difficult to detect, with a relatively stable tendency (Figure 3G). The curve of IAA and GA3 content revealed a tendency to decline at the beginning and rise in the later period, while the curve of ABA and SA content displayed a rising tendency (Figure 3A,C,E). To further understand the phytohormone fluctuations during nocturnal LTS, the percentage of phytohormones was obtained to determine the response states of *C. blinii* (Figure 3B,D,F). It was apparent in the leaves that the percentages of ABA and SA displayed a two-segment increase, slowly first and then faster (Figure 3F). During weeks 8–9, caused by the recovery of the content of IAA and GA3, an unexpected descent occurred in the percentages of ABA and SA (Figure 3F). Such a result occurred in the stems and roots as well; this process for the LTS response was indicated to be divided into three sequential segments, in which the first segment occurred in roughly weeks 1 to 4, the second segment occurred in roughly weeks 4 to 7 and the third segment occurred in roughly weeks 7 to 9.

### 3.4. The Correlation Analysis among Phytohormones, Oleanolic Acid and Blinin

**Figure 4:**
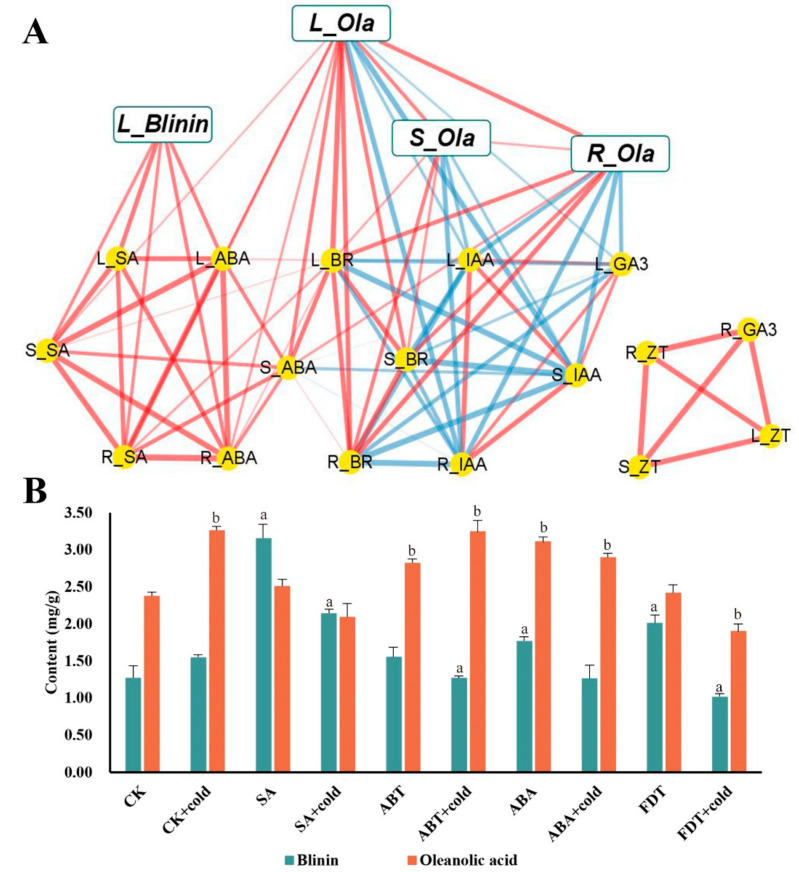
The functional verification of ABA and SA effects on terpenoid metabolism during nocturnal LTS in leaves. The correlation network among phytohormone, blinin and oleanolic acid within *C. blinii* tissues was built using Pearson ((**A**), Supplemental Tables S1 and S2). The red line is the positive correlation, while the blue line is the negative correlation. Meanwhile, The thicker and clearer the line, the stronger the correlation and the greater the *p* value. Subsequently, exogenous SA and ABA with their inhibitors were utilized to verify their function in terpenoid metabolism (**B**). Three independent strains were selected. Different letters indicate significant differences between CK and experimental groups at the same time (a, b, *p* < 0.05).

To perform a further investigation of the connection between terpenoids and phytohormones, a correlation matrix was constructed using the Pearson correlation coefficient (Supplemental Tables S1 and S2), which is illustrated in Figure 4A. Thus, it can be clearly seen that the accumulation of oleanolic acid and blinin in leaves was positively related to ABA and SA. Surprisingly, the accumulation of oleanolic acid in stems and roots showed an abundance of correlations to IAA, BR and GA3, yet lacked correlations with ABA and SA. Furthermore, as the leaves are the core site of metabolism, the accumulation of oleanolic acid was also positively correlated among tissues. To verify the function of ABA and SA, the content measurement of oleanolic acid and blinin was performed after exogenous hormone and inhibitor spraying treatment. The SA and FDT spraying groups displayed higher content of blinin (Figure 4B). In contrast, the ABA and ABT spraying groups displayed higher content of oleanolic acid.

### 3.5. Changes in Gene Expression of ABA and SA Signal Transduction Pathway in LTS

**Figure 5:**
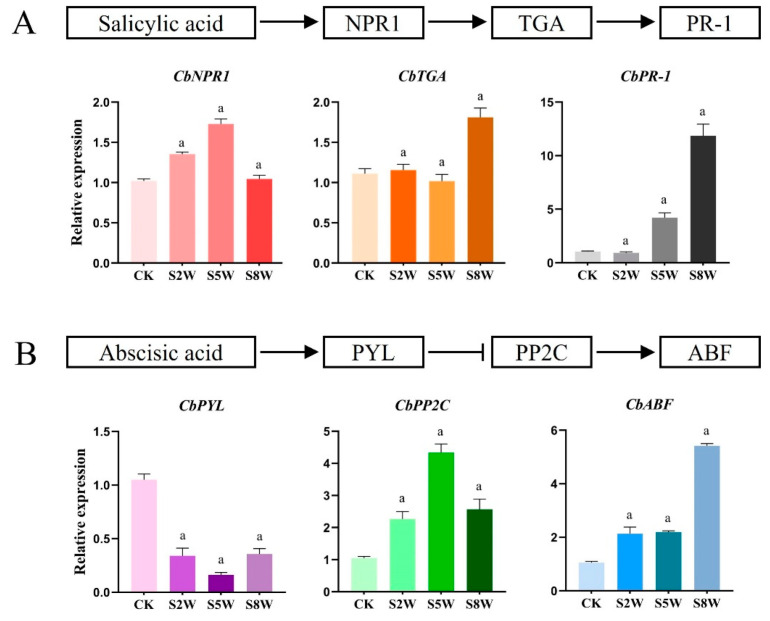
Changes in gene expression related to ABA and SA signaling pathways in LTS. (**A**) SA signaling pathway diagram and changes in expression of genes related to SA signal transduction pathway in *C. blinii*. (**B**) The schematic diagram of the ABA signaling pathway and the change in gene expression related to the ABA signal transduction pathway in *C. blinii*. CK: LTS treatment 0 weeks, S2W: LTS treatment 2 weeks, S5W: LTS treatment 5 weeks, S8W: LTS treatment 8 weeks. Three independent strains were selected. Letters indicate significant differences between CK and experimental groups at the same time (a, *p* < 0.01).

To further confirm the role of ABA and SA in LTS adaptation, we detected the gene expression of the ABA and SA pathways by qRT-PCR. It was found that the gene expression of the SA signaling pathway gradually decreased during the cold cycle. The expression of the *CbNPR1* gene was the highest in the fifth week, being approximately 1.6 times higher. The expression levels of *CbTGA* and *CbPR-1* reached the maximum in the ninth week, being, respectively, around 1.8 times and 12 times higher (Figure 5A). The expression of the *CbPYL* gene of the ABA signaling pathway decreased gradually in the cold cycle, and was the lowest in the fifth week, being approximately four times lower, indicating that the gene played an inhibitory role. The expression of the *CbPP2C* and *CbABF* genes increased gradually, reaching the maximum at the 5th and 8th weeks, respectively, being approximately four and five times higher (Figure 5B).

### 3.6. This Regulation Is Driven by Different Sources of LTS

**Figure 6:**
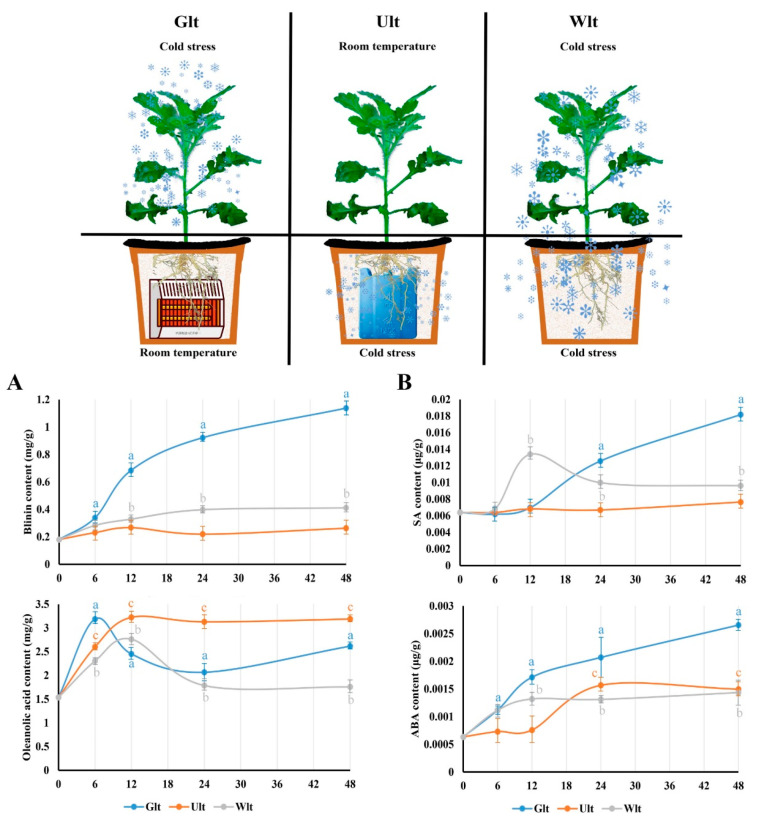
The effects of different LTS sources on blinin, oleanolic acid, SA, and ABA in leaves. In this study, the LTS source was divided into ground LTS (Glt), underground LTS (Ult), and whole LTS (Wlt) for 48 h, and we monitored the emergency trends of blinin and oleanolic acid to represent terpenoid metabolism (**A**) in leaves, as well as that of SA and ABA to represent ABA-SA crosstalk (**B**). Three independent strains were selected. Different letters indicate significant differences between CK and experimental groups at the same time (a, b, c, *p* < 0.05).

It was confirmed that LTS affected the phytohormones and terpenoids synchronously. Consequently, how did LTS drive this regulation? We broke down the sources of LTS, and divided it into ground LTS (Glt), underground LTS (Ult), and whole LTS (Wlt). As for blinin within leaves, the content increased obviously to 1.14 mg/g in Glt, while it was maintained at around 0.25 mg/g in Ult (Figure 6A). However, the content of oleanolic acid in leaves displayed an increasing tendency in each group. It should be emphasized that the content curves of Glt and Wlt both decreased after the peak value, and the decrease values were approximately 0.5 and 1.0, respectively (Figure 6A). Meanwhile, ABA and SA displayed a synchronous change in terpenoids within the leaves. Firstly, the content of ABA in Glt, Ult and Wlt significantly increased approximately 3.2-, 1.3- and 1.4-fold, respectively (Figure 6B), while the increase in Ult was delayed. In general, the content of ABA increased faster than SA driven by LTS. The content of SA in Glt, Ult and Wlt significantly increased approximately 1.8-, 0.2- and 0.5-fold, respectively, while there was a slight decline in Wlt (Figure 6B). 

## 4. Discussion

In this study, it was found that phytohormones, as a linking factor, connect LTS and terpenoid metabolism to form a “cumulative causation” within *C. blinii*. Due to the glacial–interglacial cycle, sudden and extreme LTS may occur anywhere and at any time, such as the “Younger Dryas”. Therefore, investigating the relationship among phytohormones, terpenoids, and LTS has particular significance.

### 4.1. C. blinii Planned Serial and Positive Changes in Phytohormones under Nocturnal LTS

It was found that phenotypical development was suppressed with LTS, especially in terms of stem extension and leaf and GT development. Naturally, to upgrade stress resistance, the dynamic energy flow changes from growth and development to basic and secondary metabolism. Similarly to the way in which hibernating animals store fat, *C. blinii* accumulated a large amount of photosynthetic pigment at the initial stage to prepare sufficient energy by maintaining the biosynthesis of sugar substances such as cellulose and pectin (Figure 1D). Regarding the development strategy, phytohormone is the major operator. Even at a low level, ABA was reported to act in an independent manner in LTS, responding to the regulation of COR genes, which is equivalent to the CBF-dependent pathway [25]. Based on the three-segment situation in the ABA and SA values, it was inferred that this process could be considered a procedural instinct that consists of stress perception, signal amplification, and stress adaptation in *C. blinii* when subjected to nocturnal LTS (Figure 3B,D,F). In other words, the 1st to 4th weeks were the initial stage at which the ABA and SA signaling pathways started to operate at a slowly rising percentage. With genetic memory, knowing that deep winter was approaching, *C. blinii* needed to boot all cold-related stress defense systems, so that the proportion of ABA and SA increased rapidly, while IAA and GA3 decreased rapidly, and then *C. blinii* entered a silent state to ensure a reduction of energy consumption from 4th to 7th weeks. Finally, the content of IAA and GA3 increased after the 7th week and the plant seemed to deploy a forward projection for growth and development, with a dynamic equilibrium in the content of ABA and SA (Figure 3E). 

### 4.2. The Potential Crosstalk of ABA and SA When Subjected to LTS

**Figure 7:**
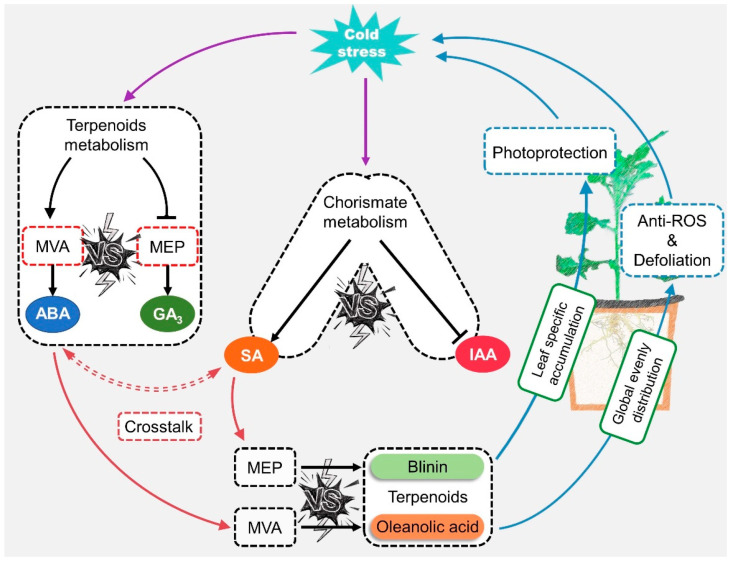
The potential mode of ABA-SA crosstalk involved in terpenoid metabolism regulation when *C. blinii* subjected to LTS.

First of all, the role of ABA and SA signals in response to LTS was judged at the level of gene transcription at three change stages in the 2nd, 5th, and 8th weeks. The results were consistent with those of other reports stating that ABA performed a dual operation in the activation of LTS defense and the inhibition of gibberellin and auxin signaling via the PP2C—SnRK—ABF” cascade [4,5]. ABA might regulate the biosynthesis of oleanolic acid and blinin in a feedback manner, and SA might play a synergistic role with biosynthesis via a distant pathway (Figure 7). Based on the evident content fluctuation (Figure 2D and Figure 3E,F), it could be inferred that the accumulation of oleanolic acid is dominated by ABA-feedback acting upon the MVA pathway. Additionally, Huang [26] reported that ABA exceeding 100 μM facilitates the accumulation of wilforlide A and represses that of triptolide and triptophenolide. AtNPR1 plays an important role in cold adaptation by regulating cold-induced gene expression independently of salicylic acid and TGA factors [27]. In this study, diterpene blinin was accumulated, with a stable increase in SA content within leaves (Figure 2B,D,E and Figure 3F). This differed from the reports that SA enhances the biosynthesis of sesquiterpenes and triterpenes via increasing gene expression in the MVA pathway [28,29]. Here, a potential crosstalk was proposed in that SA changed the metabolic flux to diterpene blinin by restricting the ABA content and signaling at a plateau after the 5th week (Figure 3E). Until the metabolic flux competition between the biosynthesis of ABA and blinin was reallocated by SA, the blinin content reached a balance with oleanolic acid gradually. Thus, ABA might function as an accelerator in MVA and an inhibitor in MEP in a concentration-dependent manner, while SA might function in a synergistic role to avoid excessive ABA-response defense. This crosstalk should be supported by the synergy and antagonism between ABA and SA [30,31,32]. Subsequently, exogenous hormone and inhibitor experiments confirmed that ABA and SA have a sufficient ability to provide a check and balance between oleanolic acid and blinin in an independent manner (Figure 4B). 

### 4.3. LTS Might Only Drive Blinii Synthesis, but Simultaneously Drive Oleanolic Acid Synthesis and Degradation

After dividing the LTS sources, Glt and Wlt were found to accumulate blinin and SA in leaves, while Ult did not. This appeared, to indicate an orientation drive system in which only ground LTS triggered this “SA—blinin” response. Firstly, it was clearly found that the transduction from the rhizosphere of SA signaling is weaker than that of ABA, particularly in Ult (Figure 6). Meanwhile, an antagonistic crosstalk exists that does not only control the biosynthesis of ABA and SA, but also balances the ABA- and SA-mediated signaling [32,33], which differed from the result of the correlation analysis (Figure 4). This may be caused by such factors as the timescale of LTS and species-specific factors. According to KEGG map01070, SA biosynthesis survives well in the competition among tryptophan, phenylalanine, tyrosine, and siderophore groups in non-ribosomal peptide metabolism. This is most probably due to the sufficient energy and substrates in the leaves, which ensures SA biosynthesis, as well as blinin from the MEP pathway. Therefore, a comprehensive study of SA metabolism and signal transduction is the key to unfolding the stress-driving mode of the SA response in metabolic regulation. On the other hand, blinin accumulated specifically in leaves might be involved in the photoprotection induced by low-temperature photoinhibition [19,34]. Furthermore, since ABA responds rapidly in the early stage and ignores the LTS source, the process of blinin accumulation displays a similar tendency to that of nocturnal LTS, which is slow at first and then becomes more rapid. Although oleanolic acid displayed rapid accumulation, it maintained stable content only in Ult while a deficit occurred in Glt and Wlt. In this context, one of the aspects that might be worth considering about is where the oleanolic acid is transported, driven by LTS. The natural world is rich in natural products for oxidation resistance, and most oleanane saponins have been proven to be capable of scavenging reactive oxygen species in vivo and in vitro [35,36,37]. Therefore, the potential degradation of oleanolic acid might occur in downstream metabolism, along with orientation driven by ground LTS. 

## 5. Conclusions

In conclusion, we observed the nocturnal low-temperature response pathway of ABA and SA terpenoid metabolism in *C. blinii* by simulating nocturnal low-temperature and local low-temperature experiments. The ABA and SA signals of *C. blinii* can participate in the MVA/MEP pathway to regulate the synthesis of terpenoids, so as to help plants adapt to the environmental changes caused by low-temperature stress.

## Data Availability

Not applicable.

## Supplementary Materials

The following supporting information can be downloaded at: https://www.mdpi.com/article/10.3390/life13020371/s1, Table S1: Pearson correlation coefficient between phytohormone content and terpenoid content; Table S2: *p* value of Pearson correlation coefficient between phytohormone content and terpenoid content.
